# Eye Symptoms in Disseminated Sclerosis

**Published:** 1905-09-09

**Authors:** 


### EYE SYMPTOMS IN DISSEMINATED SCLEROSIS.

In an important article Professor W. Uhthoff[1] relates his experience in regard to the occurrence of eye-symptoms in disseminated sclerosis. His statements are based on personal observation of 150 cases. The most important symptoms are changes in the nervous optic conducting apparatus, and more particularly in the optic nerve itself. In nearly half the cases atrophic change was detected in the appearance of the optic disc. This was either atrophic discoloration of the whole disc (3 to 4 per cent.), or a similar change involving solely or mainly the temporal half (about 20 per cent.). In 5 per cent. of all the cases optic neuritis was noted, and neuritic changes in the papilla were observed in some of the examples of optic atrophy. A further 5 per cent. showed disturbances of sight without appreciable ophthalmoscopic changes, so that altogether changes in the optic conducting apparatus were recorded in fully half of the cases of the series.

The alterations in the field of vision were as follows:—(1) The occurrence of central scotomata with free peripheral vision, the scotomata being more often relative than absolute, and usually, but not always, bi-lateral. (2) Central scotomata together with peripheral contraction of the fields (rare). (3) Peripheral but irregular contraction of the fields. (4) In isolated cases concentric functional contraction, this being observed both with, and apart from, the presence of hysteria. (5) In one case a ring-scotoma was detected. (6) Occasionally an abnormality—*e.g.* a central scotoma was observed which cleared up and was replaced by another defect—*e.g.* a peripheral contraction. (7) Hemianopic anomalies were exceptional, though the chiasma and the optic tract are recognised as frequent sites of sclerotic change. In many cases the visual disturbances were of sudden onset and later showed improvement, proving the condition was not dependent on a simple degeneration of the nerve fibres. In others the onset was more gradual, and in both groups the defect sometimes involved one and at other times both eyes. Permanent bi-lateral blindness occurred only in one case. Transitory blindness, usually in one eye, is more common, and in these cases a neuritis is frequent. Such a condition may clear up and may recur after an interval of years. Disturbances of colour vision are often insignificant, and their detection may need a very critical examination.

The condition of the eye muscles is often of importance in the diagnosis of disseminated sclerosis. The disturbances to be observed are (1) ocular paralyses and (2) nystagmus and nystagmus-like twitchings. Actual manifest paralyses were observed in 2 per cent. of the cases, but slighter grades of weakness, more particularly in the lateral movements of the eyes, were much more common. For the most part the paralyses were quite transitory. They depend usually on a central lesion, and only occasionally on defect in the peripheral nerve apparatus. The sixth nerve is the one usually involved, the third in some of its branches less frequently. Complete ophthalmoplegia externa on both sides was observed in two cases. Definite nystagmus was recorded in about 12 per cent. of the cases; it is the more important to diagnosis seeing it is a relatively infrequent symptom in other forms of organic nervous disease. Nystagmus-like twitchings are considerably more frequent, and are of greatly inferior diagnostic value. They are, too, it must be remembered, met with in other diseases, and may be seen even in persons in perfect health.

The pupils in multiple sclerosis offer few changes of clinical importance. Myosis, with very slight light reaction, was noted in 5 per cent. of the cases, a pronounced difference in the size of the pupils in 4 per cent. Complete loss of light reflex with myosis is most exceptional. Sometimes a pupil abnormally active to light is seen, but such a condition, in the first place, is difficult to define with confidence, and, secondly, is seen in other conditions, such as hysteria, neurasthenia, and epilepsy. Its diagnostic value, therefore, is slight. Oscillation of the pupil and disturbances of the functions of such nerves as the trigeminal, facial, and of the sympathetic in the neighbourhood of the eye sometimes occur, but are of little diagnostic moment.

The observation of the eye-symptoms in disseminated sclerosis is of great value, and more particularly where the clinical evidences of the disease are limited to the lower limbs, and where, therefore, the suggestion is that the case is one of a purely spinal nature. The recognition of such changes as are above described at once, in such instances, compels the enlargement of the diagnosis so as to include the existence of cerebral changes.

[1] The Ophthalmoscope, September 1905.

